# Artificial intelligence sepsis prediction algorithm learns to say “I don’t know”

**DOI:** 10.1038/s41746-021-00504-6

**Published:** 2021-09-09

**Authors:** Supreeth P. Shashikumar, Gabriel Wardi, Atul Malhotra, Shamim Nemati

**Affiliations:** 1 Division of Biomedical Informatics, University of California San Diego, San Diego, USA; 2 Department of Emergency Medicine, University of California San Diego, San Diego, USA; 3 Division of Pulmonary, Critical Care and Sleep Medicine, University of California San Diego, San Diego, USA

**Keywords:** Predictive medicine, Computational models, Machine learning, Bacterial infection, Translational research

## Abstract

Sepsis is a leading cause of morbidity and mortality worldwide. Early identification of sepsis is important as it allows timely administration of potentially life-saving resuscitation and antimicrobial therapy. We present COMPOSER (COnformal Multidimensional Prediction Of SEpsis Risk), a deep learning model for the early prediction of sepsis, specifically designed to reduce false alarms by detecting unfamiliar patients/situations arising from erroneous data, missingness, distributional shift and data drifts. COMPOSER flags these unfamiliar cases as *indeterminate* rather than making spurious predictions. Six patient cohorts (515,720 patients) curated from two healthcare systems in the United States across intensive care units (ICU) and emergency departments (ED) were used to train and externally and temporally validate this model. In a sequential prediction setting, COMPOSER achieved a consistently high area under the curve (AUC) (ICU: 0.925–0.953; ED: 0.938–0.945). Out of over 6 million prediction windows roughly 20% and 8% were identified as *indeterminate* amongst non-septic and septic patients, respectively. COMPOSER provided early warning within a clinically actionable timeframe (ICU: 12.2 [3.2 22.8] and ED: 2.1 [0.8 4.5] hours prior to first antibiotics order) across all six cohorts, thus allowing for identification and prioritization of patients at high risk for sepsis.

## Introduction

Sepsis is a dysregulated host response to infection causing life-threatening organ dysfunction^1^. Approximately one in three hospital deaths are attributable to sepsis^2^. While effective protocols exist for treating sepsis^3^, challenges remain in early and reliable detection of this condition^4^. In recent years, the increased adoption of electronic medical records (EHRs) in hospitals has motivated the development of machine learning-based surveillance tools for detection^5–9^ and prediction^10–15^ of sepsis. However, most existing published sepsis prediction models^16,17^ are either based on data from a single hospital^5,7–10,13,15,18,19^ or multiple hospitals from the same healthcare system^11,14^ where the care processes are mostly standardized. Three major barriers to the regulatory approval^20^ and widespread adoption of these systems are (1) lack of generalizability across institutions, (2) high false alarm rates, and (3) risk of *automation bias*, wherein users tend to over-rely on the system output instead of active information seeking and risk assessment^21,22^. One of the main factors contributing to an algorithm’s performance degradation (including increased false alarm and missed-detection rate) across sites is the data distribution shift (encountering unfamiliar patients) and variations in levels of data missingness caused by differences in hospital workflow and practices^23,24^. Moreover, a recent study demonstrated that detecting outlier cases and showing users an outlier focused message better enabled them to detect and correct for potential spurious predictions by an AI model^25^. However, while recent literature has emphasized the importance of including clear ‘indication of use’ labels with machine learning algorithms^26^, none of the existing sepsis prediction algorithms include a built-in mechanism for detecting outliers and for establishing the ‘condition for use’ of the model across geographical and temporal domains.

**Fig. 1:**
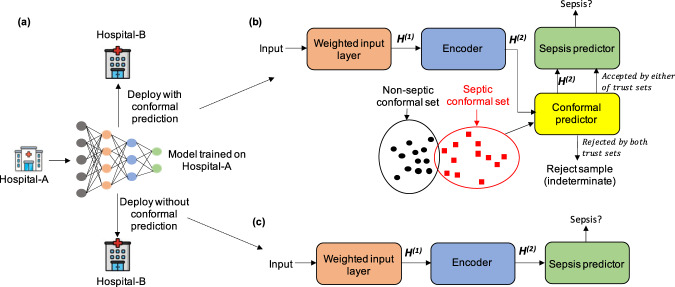
Schematic diagram of COMPOSER. Two possible deployment schemes are shown in panel (**a**). During the evaluation phase of COMPOSER (panel **b**), the input test data is first fed into the weighted input layer, and is then passed through the encoder (*first module*), after which the method of conformal prediction is utilized to determine the ‘conditions for use’ (*second module*). This is achieved by comparing the conformity of the new representation (*H*^(2)^) to the representations in the conformal set, which are carefully selected during the training phase (see Methods section). If conformity can be achieved at a given confidence level (ε), *H*^(2)^ is then forwarded to the sepsis predictor to obtain a risk score (*third module*). In comparison, panel (**c**) shows a deployment scheme without the use of conformal prediction.

In this work, we propose COMPOSER (Conformal Multidimension Prediction of Sepsis Risk), a deep learning model designed to predict onset of sepsis 4–48 h prior to time of clinical suspicion. COMPOSER achieves improved generalizability and low false alarm rates through a prediction scheme that statistically determines conformity with a predefined collection of representations (aka *conformal set*), as a means to establish the ‘conditions for use’ of the algorithm under unseen prediction scenarios including new patient populations and different levels of data quality and missingness. The proposed COMPOSER model consists of three modules. The *first module* makes use of clinical variables and timing information about measurements to generate lower dimensional representations that are robust to patterns of data missingness and institution-specific workflow practices^27,28^. The *second module* includes a *conformal prediction*^29–32^ network, which provides a statistical framework for detecting out-of-distribution (i.e., *indeterminate*) samples during the risk assessment phase in a deployment environment. Two bags of data representations (aka, *conformal sets*) are used to quantify explicitly the conformity of new patient-level feature vectors to the previously seen examples of septic and non-septic feature vectors within the development cohort. The conformal prediction allows the model to detect outlier inputs that do not satisfy the conditions for use of the algorithm, which are subsequently assigned to an *indeterminate* predicted label class. Supplementary Fig. 3 provides an illustration of scenarios under which a test sample is accepted or rejected by the conformal prediction module. The *third module* includes a sepsis predictor that is a feedforward neural network followed by a logistic regression. Figure 1b provides the overall schematic diagram of COMPOSER during the evaluation phase.

## Results

### Study population and evaluation

COMPOSER was trained and evaluated on six patient cohorts (515,720 patients) across two academic medical centers (Hospital A, Hospital B) in the US, including three ICU cohorts and three ED cohorts collected between 2016 and 2020. The patient characteristics of all the six cohorts have been tabulated in Supplementary Tables 2 and 3. Patients in the Hospital-A ICU and ED cohorts were randomized across training (80%) and testing (20%) sets. The entire Hospital-A Temporal ICU and ED, and Hospital-B ICU and ED cohorts were used for temporal and external validation respectively. Patients 18 years or older were followed throughout their stay until time of first episode of sepsis or otherwise time of transfer out of their current unit (ICU or ED depending on the cohort). Sepsis was defined according to the latest International Consensus Definitions for Sepsis (Sepsis-3)^1^^,33^.

More details about the development of COMPOSER can be found in the Methods section. We evaluate the performance of this model on six patient cohorts, using a modified Area Under the Curve (AUC) metric evaluated under a clinically relevant protocol, as described by Hyland et al.^34^ More Specifically, AUCs are reported under an end-user clinical response policy in which the model was silenced for 6 h after an alarm was fired, and alarms fired 4–48 h prior to onset of sepsis were considered as true alarms. Additionally, we report threshold-based performance metrics (at a fixed sensitivity of 80%), with a focus on false alarms and misdetections, using positive predictive value (PPV) and negative predictive value (NPV), respectively.

### Internal testing set

COMPOSER’s performance (AUC/PPV) on the source cohort ICU and ED testing dataset was 0.953/38% and 0.945/20.1%, respectively. COMPOSER was able to achieve this performance while maintaining low false alarms (false alarms per patient hour of 0.031 and 0.042; SPC of 93.0% and 93.5%). Supplementary Figs. 6 and 7 show heatmaps of the top 15 variables contributing to the increase in risk score up to 12 h prior to onset of sepsis. It was observed that clinical variables related to sepsis (e.g., temperature, white blood count, heart rate) were identified as the top contributing factors.

### External validation

**Fig. 2:**
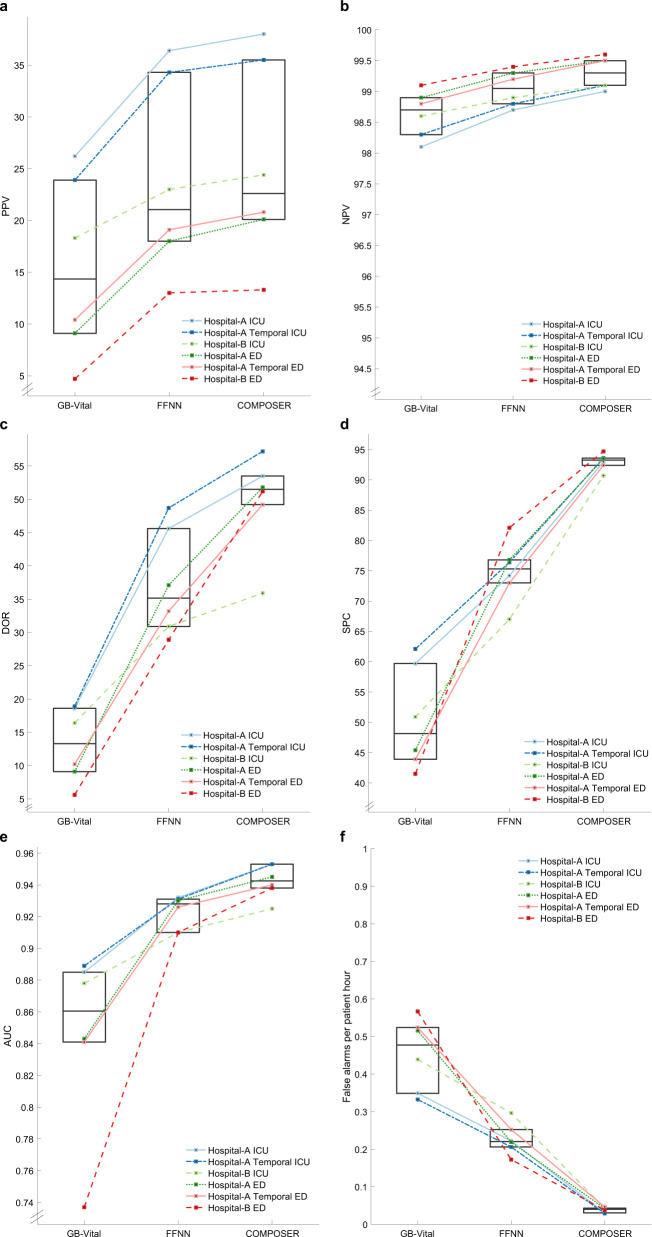
Summary of COMPOSER performance. Comparison of COMPOSER model against *GB-Vital*^a^ and a feedforward neural network (*FFNN*^b^). The line plots in **a**–**f** shows the relative improvement in positive predictive value (PPV), negative predictive value (NPV), diagnostic odds ratio (DOR), specificity (SPC), Area Under the Curve (AUC) and number of false alarms per patient hour (FAPH)^+^, respectively. The median and interquartiles for all six cohorts (three ICUs and three EDs) are summarized via superimposed box plots. In comparison, *ESPM*^*c*^ (not shown here) achieved an AUC of 0.889 (PPV = 31.2%, NPV = 97.8%, DOR = 23.2, SPC = 84.3, FAPH = 0.132) and 0.876 (PPV = 35.9%, NPV = 96.8%, DOR = 17.4, SPC = 94.2%, FAPH = 0.05) across Hospital-A temporal ICU and ED. ^a^*GB-Vital* corresponds to a Gradient Boosted Tree (XGBoost)^6,15^ built using six vital signs measurements: systolic blood pressure, diastolic blood pressure, heart rate, respiratory rate, oxygen saturation and temperature. ^b^*FFNN* corresponds to a 2 layer feedforward neural network that uses the same number of input features as that of COMPOSER. The starting point of y-axis for **a** and **b** were determined by the chance level of a classifier at the lowest prevalence rate. ^c^ESPM corresponds to the Epic’s commercially available Best Practice Advisory (BPA) alert. We only had access to the risk scores produced by this system at Hospital-A during the temporal validation time-frame. ^+^ False alarms per patient hour (FAPH) can be used to calculate the expected number of false alarms per unit of time in a typical care unit (e.g., a FAPH of 0.025 translates to roughly 1 alarm every 2 h in a 20-bed care unit).

When applying COMPOSER to data from Hospital-B ICU patients, compared to a baseline feedforward neural network (FFNN) model, COMPOSER achieved roughly 85.5% relative reduction in false alarms (FAPH of 0.043 versus 0.296; SPC of 90.7% vs 67.0%) while maintaining superior AUC and PPV (AUC of 0.925 vs 0.910, *p* < 0.001; PPV of 24.3% vs 23.0%). Similarly, within the Hospital-B ED cohort COMPOSER achieved roughly 77.9% relative reduction in false alarms (FAPH of 0.038 versus 0.172; SPC of 94.7% vs 82.1%) while maintaining superior AUC and PPV (AUC of 0.938 vs 0.910, *p* < 0.001; PPV of 13.4% vs 13.0%). See Fig. 2a–f for a comparison of COMPOSER’s performance (PPV, NPV, DOR, SPC, AUC, and False alarms per patient hour) vs the baseline FFNN model across all cohorts.

### Outlier detection and indeterminates

Overall 75–86% of the prediction windows (see Supplementary Table 10 and Supplementary Table 11) satisfied the conditions for use of the algorithm. However, within the septic windows the percentage of *indeterminate* cases were almost half compared to the non-septic windows (24.6–27.8% versus 12.4–13.9% for the ICU cohorts and 13.6–16.1% versus 6.5–9.9% for the ED cohorts). Moreover, patient-wise analysis revealed that the median percentage of septic patients with all septic windows rejected was 1.1% [0.87–2.6% IQR], indicating that conformal prediction had minimal deleterious effect on the patient-wise sensitivity of the algorithm.

### Temporal validation

To assess the impact of changes in institutional practices and patient populations over time we performed an experiment in which a model trained on a combined cohort of ICU and ED patients (which consisted of patients admitted from January 2016 through March 2019) from Hospital-A was applied to a temporal validation set consisting of patients admitted to Hospital-A from August 2019 through February 2020. COMPOSER achieved an AUC of 0.940 (PPV of 20.8%) on the Hospital-A Temporal ED validation cohort and an AUC of 0.952 (PPV of 35.5%) on the Hospital-A Temporal ICU validation cohort (see Table 2). COMPOSER was able to achieve this performance while maintaining low false alarms; false alarms per patient hour of 0.047 (SPC of 92.4%) and 0.029 (SPC of 93.6%) on the temporal ED and ICU cohorts, respectively. This finding suggested that the model was not adversely impacted by changes in patient population or clinical practices over time. Additionally, COMPOSER achieved roughly 78% relative reduction in false alarms in comparison to a commercially available sepsis prediction model (ESPM) in use at Hospital-A during the same time period (FAPH of 0.132 vs 0.029) in the ICU. Moreover, COMPOSER achieved significantly better alarm notification lead-time, in advance of clinical suspicion of sepsis, compared to the ESPM model (see Table 1).

**Table 1:** Time from model prediction to clinical suspicion of infection.

	Δ*t*_*lactate*_ (in hours)	Δ*t*_*suspicion*_ (in hours)	Δ*t*_*ABX*_ (in hours)	Δ*t*_*culture*_ (in hours)
Hospital-A Temporal ICU				
COMPOSER	2 [0 7]	8.4 [1.2 11.5]	12.2 [3.2 22.8]	11.2 [5.2 11.9]
ESPM (score>5)	1 [0 5]	3.5 [0.2 11.2]	10.2 [1.0 18.9]	8.9 [1.2 11.9]
Hospital-A Temporal ED				
COMPOSER	1 [1 2]	1.0 [0.3 2.3]	2.1 [0.8 4.5]	0.8 [0.2 3.4]
ESPM (score>5)	0 [−1 1]	0.1 [−0.9 0.65]	0.6 [0.2 2.0]	0.2 [−0.8 1.0]

The difference between the time at which an alarm (*t*_*alarm*_) was fired (i.e., risk score exceeded a decision threshold) and *t*_*lactate*_ */t*_*suspicion*_*/t*_*ABX*_*/t*_*culture*_ for the Hospital-A Temporal ICU and ED cohorts are shown. *t*_*alarm*_ corresponded to the earliest time at which an alarm was fired in the interval [*t*_*suspicion*_˗ 12 h, *t*_*suspicion*_+ 12 h]. *t*_*lactate*_ corresponded to the earliest time at which a lactate measurement was made in the interval [*t*_*suspicion*_˗ 12 h, *t*_*suspicion*_+ 12 h]. *t*_*ABX*_ corresponded to the time at which antibiotics were ordered following *t*_*suspicion*_. *t*_*culture*_ corresponded to the time at which cultures were ordered following *t*_*suspicion*_. A negative value indicates the alarm was fired after the time point of interest.

### Analysis of missed-detections

We observed that COMPOSER achieved high negative predictive value across all the six cohorts (98.8–99.1% in ICU and 99.5–99.6% in ED) when evaluated in a sequential prediction setting. Additionally, it was also observed that COMPOSER made at least one positive prediction within the septic window in 91.3–92.3% and 90.5–95.6% of septic patients in the ICU and ED cohorts respectively. In other words, COMPOSER’s missed-detection rate amongst septic patients was below 10% across all the six cohorts. Thus mitigating concerns about deleterious effects of automation bias; in particular, when used in association with the standard of care and existing hospital workflow practices for identification of sepsis.

## Discussion

We presented a generalizable deep learning model for the continuous prediction of sepsis within a clinically actionable window of 4–48 h in advance of clinical suspicion. Using representation learning and conformal prediction allowed us to introduce a formal procedure to detect outlier inputs (or unfamiliar patients/situations) arising from erroneous data, missingness, distributional shift and data drifts, and thus establishing the ‘conditions for use’ of the algorithm. COMPOSER was designed to be locally interpretable wherein the model was capable of identifying the most relevant features contributing positively or negatively to the increase in risk score at every point in a patient’s timeline. Finally, we show consistent performance across different levels of care, hospitals, and retrospective and temporal validation cohorts, demonstrating that our approach is broadly applicable to different care settings.

By carefully constructing two conformal sets for the representation of septic and non-septic data points by, for example, varying tolerance for missing data, conformal prediction was able to control the rejection rate of septic and non-septic cases independently. As such, labeling outlier inputs as *indeterminate* resulted in relative reduction in the rate of false alarms in the range 77–85% compared to baseline models, while keeping sepsis missed-detection rates among the *indeterminate* cases close to zero. In addition to the improved PPV, COMPOSER’s NPV remains high (98.8% ICU and 99.5% ED), thus mitigating concerns around potential missed-detections and automation bias when used in parallel with the standard of care for detection of sepsis.

Both false positives and false negatives are cited by the U.S. Food and Drug Administration as key factors for consideration when making benefit-risk determination in Software as a Medical Device (SaMD) Premarket Approvals and De Novo Classification^35,36^. While false alarms have the potential to increase caregiver’s cognitive burden and may expose patients to unnecessary tests and treatments^37^, missed detections can also cause harm if the potential of automation bias is not taken into account during the implementation phase of an algorithm. For example, a centralized implementation (‘command control’)^18^ which treats the algorithm as a second ‘pair of eyes’, when deployed in parallel to standard of care, may mitigate some of the concerns surrounding automation bias^20,38^. Nevertheless, bedside implementation of such algorithms pose additional challenges. For instance, higher than usual levels of data missingness may result in a low score in a patient not exhibiting overt signs of sepsis, which may create a false sense of confidence. Detection of low quality data (e.g., due to high levels of data missingness) and flagging them as indeterminate may reduce potential harm caused by automation bias.

Recent literature has emphasized the importance of including information on ‘uses and warnings’ with machine learning models, which provide information such as model name, locale, version, summary of the model, mechanism of risk score calculation, validation and performance, uses and directions, warnings, and other information^26^. However, to date, none of the existing sepsis prediction algorithms include a built-in mechanism for detection of outliers and for establishing the ‘condition for use’ of the model across geographical and temporal domains. As currently there are no legally marketed predicate machine learning-based sepsis prediction SaMDs in the market^36^, there is an unmet need for design of quality control criteria for clinical implementation and successful regulatory approval of such algorithms. COMPOSER utilizes a conformal prediction framework to explicitly quantify the conformity of new patient-level feature vectors to the previously seen examples of septic and non-septic feature vectors within the development cohort. As such, the proposed approach complements and extends the ‘intended use’^39^ and ‘model fact labels’^26^ of a machine learning algorithm, and provides a statistical approach for detecting outliers and out-of-distribution data at a finer resolution.

While we have demonstrated that flagging of indeterminate cases results in significant reduction in false alarms, it is not readily obvious how these indeterminate cases should be treated differently by the end-user compared to episodes of low risk score. To improve actionability, one may distinguish among different categories of indeterminates as a component of an alarm verification process^40^. Such categories may include indeterminate cases with low vs. high risk scores, as well as, those with low vs. high level of data missingness. Each such scenario may result in an actionable set of recommendations (e.g., ordering of additional labs). Furthermore, weekly or monthly statistics of rates of indeterminacy can be used as a trigger to activate an algorithm ‘change protocol plan’^41,42^ in a systematic manner that manages risks to patients (e.g., fine-tuning of the model on the target population^43^). Nevertheless, recent literature demonstrates that even simple flagging of outlier cases in complex machine learning models (see Figure S10) is likely to enable the end-users to detect and correct for potential model mistakes^25^.

Any algorithm designed to monitor longitudinally a rare event (e.g., hourly prediction of sepsis) is bound to suffer from some level of false alarms. Moreover, the gold-standard labels for sepsis have limited temporal resolutions and the presence of competing risk factors induce additional levels of diagnostic uncertainty. We observed that roughly 50% of COMPOSER’s false alarms were triggered on patients who satisfied at least one of the following six conditions within 72 h of a false alarm: (1) presence of clinical suspicion of infection without evidence of acute organ dysfunction, (2) need for vasopressors, (3) needing mechanical ventilation, (4) at risk for acute kidney injury, (5) eventual transition to sepsis, but not within 48 h of the alarms, and (6) risk for mortality or needing hospice care (see Supplementary Table 8). As such, improvements in differential prediction and prognostication are needed to enhance further the actionability of such alarms.

In this work, due to limited availability of data we only focused on ED and ICU care units. As such the current algorithm is not optimized for non-ICU inpatient wards. It is known that the frequency of EHR measurements is commonly a function of care levels (e.g., emergency departments, ICUs, step-down units/general wards, long-term care facilities, and nursing homes), workflow practices, and patients’ severity of illness^27^. This situation in turn has resulted in ‘data deserts’ in some settings and ‘data deluge’ in others^44^. To maximize predictive performance within different care settings, input to such algorithms could be enriched with higher resolution data from wearable sensors, bedside monitors and biomarkers for pathogen profiling and host inflammatory response to infection. The proposed algorithm provides a foundational building block for the design of future generalizable and trustworthy prediction tools potentially useful in other clinical syndromes and disease processes. A real-time HL7 FHIR^45,46^ compatible software pipeline has been developed to enable interoperable multi-center deployment of this algorithm (See Supplementary Note 10 for more details). Future work includes performing prospective clinical trials to validate COMPOSER’s predictions in a real-time clinical setting. However, our findings provide significant clinical evidence for a radical improvement in early identification of sepsis, with significantly lower false alarm rates, and has the potential to improve sepsis-related clinical outcomes.

## Methods

### Dataset description

We collected de-identified data from the EHR across different care-levels and time-frames from two academic medical centers, the University of California San Diego Health and Emory University Hospital, in the United States to construct a total of six patient cohorts (total of 515,720 encounters). Throughout the manuscript, we refer to the respective hospital systems as Hospital-A and Hospital-B. Supplementary Tables 2 and 3 contain detailed information regarding the six cohorts considered in this study. Patients 18 years or older were followed throughout their stay until time of first episode of sepsis or otherwise time of transfer out of a given care unit (ICU or ED depending on the cohort). To allow for initial examination and stabilization of patients and adequate data collection for prediction purposes, we focused on sequential hourly prediction of sepsis starting at hours two and four within our ED and ICU cohorts, respectively. Patients who were identified as having sepsis prior to prediction start time or those with no measurement of heart rate or blood pressure prior to the prediction start time or those whose length of stay (ICU or ED depending on the cohort) was more than 21 days were excluded.

The overall dataset was divided into the following cohorts: (1) *training cohort* (80% of encounters from Hospital A ICUs and EDs, including over 13,000 ICU encounters and over 79,000 ED encounters), (2) *testing cohort* (20% of the encounters from Hospital A ICUs and EDs), (3) *temporal validation cohort* (prospectively collected encounters from Hospital A ICUs and EDs), and (4) *external validation cohort* (retrospective data from Hospital B ICUs and EDs). See Table 2 for a breakdown of patients used in cohorts 2–4 for evaluation purposes after the model was trained on cohort 1 and was frozen. The Hospital-A training and testing cohorts were collected between January 2016 and August 2019, and the temporal validation cohort was collected between August 2019 through February 2020. The external validation cohort was collected between January 2014 to December 2018.

**Table 2:** Summary of performance of COMPOSER on the (A) ICU and (B) ED cohorts when evaluated in a sequential prediction setting.

	SEN	SPC	PPV	NPV	DOR	AUC
(A) ICU cohorts						
Hospital-A ICU test N^a^=3,406; S^a^=767 (22.5%)	91.6^a^	93.0^b^	38.0^b^	98.8^b^	53.5^b^ (1.088)	0.953
N^b^=157,527; S^b^=6499 (4.1%)	80.1^b^					
Hospital-A Temporal ICU N^a^=3,596; S^a^=733 (20.4%)	92.3^a^	93.6^b^	35.5^b^	99.0^b^	57.2^b^ (1.090)	0.953
N^b^=171,242; S^b^=6156 (3.6%)	79.3^b^					
Hospital-B ICU N^a^=45,812; S^a^=7,913 (17.3%)	91.3^a^	90.7^b^	24.3^b^	99.1^b^	35.9^b^ (1.027)	0.925
N^b^=1,884,383; S^b^=56,637(2.9%)	78.9^b^					
(B) ED cohorts						
Hospital-A ED test N^a^=19,807; S^a^=1,624 (8.2%)	95.6^a^	93.5^b^	20.1^b^	99.5^b^	51.8^b^ (1.109)	0.945
N^b^=162,504 S^b^=7691 (4.7%)	78.1^b^					
Hospital-A Temporal ED N^a^=19,945; S^a^=1,795 (9.0%)	96.0^a^	92.4^b^	20.8^b^	99.5^b^	49.2^b^ (1.107)	0.940
N^b^=158,045; S^b^=8766 (5.5%)	80.0^b^					
Hospital-B ED N^a^=330,299; S^a^=14,454 (4.4%)	90.5^a^	94.7^b^	13.4^b^	99.6^b^	51.2^b^ (1.031)	0.938
N^b^=2,362,426; S^b^=64032(2.7%)	70.5^b^					

Decision threshold corresponding to 80% sensitivity on the Hospital-A training cohort (0.561 and 0.414 for the ICU and ED cohorts respectively).

*N* Number of patients within the cohort, *S* Number of septic patients.

^a^Patient-wise.

^b^hourly window-wise.

*SEN* Sensitivity, *SPC* Specificity, *PPV* Positive predictive value, *NPV* Negative predictive value, *DOR* Diagnostic odds ratio with 95% confidence interval.

We followed the latest guidelines provided by the Third International Consensus Definitions for Sepsis (Sepsis-3)^1,33^ which defined sepsis as a life-threatening organ dysfunction caused by a dysregulated host response to infection. As such, the two main criteria for establishing onset time of sepsis included: (1) evidence of acute organ dysfunction, and (2) suspicion of infection. Clinical suspicion of infection was defined by blood culture draw and new start of intravenous (IV) antibiotics continued for > = 3 consecutive days (excluding prophylactic use) satisfying either of the following conditions: (a) if a blood culture draw was ordered first, then antibiotics order had to occur within the following 72 h, or (b) if antibiotics order occurred first, then a blood culture draw had to occur within the next 24 h. Evidence of organ dysfunction was defined as an increase in the Sequential Organ Failure Assessment (SOFA) score by two or more points. In particular, evidence of organ dysfunction occurring 48 h before to 24 h after the time of suspected infection was considered, as suggested in Seymour et al.^33^. Finally, time of onset of sepsis was taken as the time of clinical suspicion of infection.

This investigation was conducted according to University of California San Diego IRB approved protocol #191098 and Emory University IRB Protocol #00110675. A waiver of consent was granted by the IRB as this was a retrospective study.

### Model features

Consistent with the PhysioNet Sepsis Challenge 2019, a total of 40 clinical variables (34 dynamic and 6 demographic variables, see Supplementary Table 1) were extracted based on their association with the onset of sepsis and their availability in EHR across the two hospitals considered in our study^11,12,14^. These included vital signs measurements (heart rate, pulse oximetry, temperature, systolic blood pressure, mean arterial pressure, diastolic blood pressure, respiration rate, and end-tidal carbon dioxide), laboratory measurements (bicarbonate, bicarbonate excess, fraction of inspired oxygen, pH, partial pressure of carbon dioxide from arterial blood, oxygen saturation from arterial blood, asparate transaminase, blood urea nitrogen, alkaline phosphatase, calcium, chloride, creatinine, bilirubin direct, serum glucose, lactate, magnesium, phosphate, potassium, total bilirubin, troponin, hematocrit, hemoglobin, partial thromboplastin time, leukocyte count, fibrinogen and platelets) and demographic variables (age, gender, identifier for medical ICU unit, identifier for surgical ICU unit, length of hospital stay, length of ICU stay). All vital signs and laboratory variables were organized into 1-h non-overlapping time series bins to accommodate for different sampling frequencies of available data. All the variables with sampling frequencies higher than once every hour were uniformly resampled into 1-h time bins, by taking the median values if multiple measurements were available. Variables were updated hourly when new data became available; otherwise, the old values were kept (sample-and-hold interpolation). Mean imputation was used to replace all remaining missing values (mainly at the start of each record). Additionally, for every vital signs and laboratory variable, their local trends (slope of change), baseline value (mean value measured over the previous 72 h), and the time since the variable was last measured (TSLM) were recorded. Hereafter, we refer to the 34 dynamical variables and their local trends (total of 102 features) by *X*_*dynamical*_, the 34 TSLM features by *X*_*TSLM*_ and the 6 covariate features by *X*_*covar*_, resulting in a total of 108 features.

### Development of the COMPOSER model

COMPOSER consists of three modules. First, a *weighted input layer* that scales the value of a clinical variable depending on the time since it was last measured. Intuitively, this layer attempts to mimic a clinician’s thought process of putting more importance on the most updated vitals and labs, depending on the physiologically plausible rates at which such measurements can change. As such, the weighted input layer was designed to incorporate some information about the timing of clinical measurements without allowing the network to exploit the correlation between frequency of measurements and disease severity or patients’ level of care^47^. Such factors are often affected by the institution-specific workflow practices and care protocols and likely to reduce the generalizability of a predictive algorithm. The output of this layer was fed into an *encoder network* (a feed forward neural network) that is used to reduce data dimensionality. The second module is a *conformal predictor* which is used to establish the ‘conditions for use’ of the model by statistically assessing the conformity of any new test instance to a pre-constructed bag of examples (‘conformal set’) drawn from the training set. The third module includes a sepsis predictor, which is a feedforward neural network whose output is a probability score (between 0 and 1) that represents the risk of sepsis. All the modules are parametrized as neural networks and trained end-to-end, and enable the application of local interpretability methods such as relevance scores (RS) and layer-wise relevance propagation (LRP)^48^.

Conformal prediction enables the algorithm to determine the level of data distribution shift, including data quality and missingness level, at which the input data remains appropriate for prediction. The development and evaluation of COMPOSER involved two steps: First, the first and the second modules were trained using the combined Hospital-A ICU and ED training sets. Second, the conformal set (consisting of representations from the encoder module) was constructed from the combined Hospital-A ICU and ED training sets. Finally, the trained modules along with the conformal set were used for model evaluation. Each of the individual components of COMPOSER, namely the weighted input layer and conformal prediction are explained in detail in the following sections.

### Weighted input layer

We designed a weighted input layer that scales the latest measured value of a variable depending on the duration since it was measured. This scaling enables the model to appropriately account for the age of an imputed feature while constraining the model from directly exploiting the frequency of measurements. The extent of scaling is controlled by a parameter *α* that is learned from data. Let us consider $X_t^n$ to be a centered and standardized 68 dimensional vector (consisting of all dynamical variables) at time *t* for patient *n*. Henceforth, with a slight abuse of notation we will refer to $X_t^n$ by $X,$ wherein ${\mathrm{X}} = \left[ {x_1;x_2; \ldots .;x_{68}} \right],x_j \in {\mathbb{R}}$. Next *δ*_*j*_ corresponds to the duration since variable *j* was last measured (in reference to the current time *t*). Each of the variables *x*_*j*_ is then non-linearly weighted based on the duration since it was last measured, to obtain $h_j^1 = \left[ {x_j \ast f(\delta _j,\alpha _j)} \right]$. The weighting function $f(.)$ is defined as follows:

1 $$f\left( {\delta _j,\alpha _j} \right) = 2 \ast \left( {1 - \frac{1}{{\left( {1 + \exp \left( { - \alpha _j^2 \ast \frac{{\delta _j}}{{24}}} \right)} \right)}}} \right)$$

Where *α*_*j*_ is a scaling factor for each of the dynamical variables and is learned during the training of the model. Thus, the output of the weighted input layer is a 68 dimensional feature vector $H^{(1)} = \left[ {h_1^1,h_2^1, \ldots ,h_{68}^1} \right]^T$. The scaling factors *α*_*j*_ obtained at the end of model training is inversely related to the extent of sample-and-hold interpolation that is useful for the i-th variable.

### Detecting distribution shift using conformal prediction

We used the method of *conformal prediction*^29,31,32^ to develop a statistical test to determine whether a given data sample belongs to the data distribution from which the training data was drawn and model performance during training was high. A sepsis prediction is made on the data sample only if it belongs to the training distribution of COMPOSER, else the data sample is rejected and no sepsis prediction is made. Two sets of size *M* of the representations $H_i^{(2)}$ from the source dataset (*S*) each containing only septic and non-septic examples, respectively, are chosen as the trust sets $\tau ^{septic} = \{ {H_{1,septic}^{(2)},H_{2,septic}^{(2)}, \ldots ,H_{M,septic}^{(2)}} \}$ and $\tau ^{nonseptic} = \{ {H_{1,non - septic}^{(2)},H_{2,non - septic}^{(2)}, \ldots ,H_{M,non - septic}^{(2)}} \}$. These conformal sets were selected by performing a grid search over training examples based on their levels of missingness and cross entropy error (i.e., a non-septic case with high risk score and vice versa) and by choosing the cut-offs for inclusion that achieved the highest F2 score; since the cost of missing a septic event (missed detection) is considerably higher than a false alarm, the conformal set was designed to emphasize sensitivity over precision. During evaluation phase, the model was presented with a test example $H_{M + 1}^{(2)}$ for which the task of conformal prediction was (1) to predict if the test example is drawn from the same probability distribution as that of other examples in the either of the trust sets *τ*^*septic*^ and *τ*^*non-septic*^ at a given confidence level (1−*ε*), and 2) if yes, the test example $H_{M + 1}^{(2)}$ was passed onto sepsis predictor to obtain the sepsis risk score.

#### Hypothesis testing

First, we aimed to measure how likely it was that a given sequence of examples were drawn from the same probability distribution. We use the term *p-value* to measure the typicalness of a sequence of examples (H*) wherein the *p-value* was computed using a function $p\!\!:H^ {\ast} \to [0,1]$. For a given input $H_{M + 1}^{\left( 2 \right)}$ the *p-value* of $H_{M + 1}^{\left( 2 \right)}$ w.r.t *τ*^*septic*^ denoted by $p(H_{M + 1};\tau ^{septic})$ refers to typicalness of the sequence $\left( {H_{1,septic}^{\left( 2 \right)},H_{2,septic}^{\left( 2 \right)}, \ldots ,H_{M,septic}^{\left( 2 \right)},H_{M, + 1}^{\left( 2 \right)}} \right)$ (the sequence consists of all examples in conformal set $\tau ^{septic}$ plus the given test example). If *p-value* of a given test example is under some very low threshold (e.g., 0.05), this would signify that such a sequence would only be generated at most 5% of the time by any i.i.d process, and is unlikely to belong to the probability distribution of the conformal set. In other words, the hypothesis being tested says “All examples in the sequence $\left( {H_{1,septic}^{(2)},H_{2,septic}^{(2)}, \ldots ,H_{M,septic}^{\left( 2 \right)},H_{M, + 1}^{(2)}} \right)$ belong to the same probability distribution”, and the hypothesis is rejected if $p(H_{M + 1};\tau ^{septic}) \le \varepsilon$ for some predetermined *ε*. The same procedure is repeated w.r.t *τ*^*non-septic*^ wherein $p(H_{M + 1};\tau ^{non - septic})$ is computed and the same hypothesis test is performed.

The *p-value* function can be constructed by comparing how different each example in the sequence is from all the other examples. This is possible using the measure of nonconformity. The measure of nonconformity intuitively corresponds to how atypical a sequence is, and maps a bag of examples and one additional example to a scalar ${\upeta}_i \in R$:

2 $$\upeta _{\mathrm{i}} = A\left( {\left\{ {H_1^{(2)}, \ldots ,H_{i - 1}^{\left( 2 \right)},H_{i + 1}^{(2)} \ldots ,H_{M + 1}^{(2)}} \right\},H_i^{(2)}} \right)$$

for each example $H_i^{(2)}$, thereby measuring how different it is from other examples in the bag $\{ {H_1^{(2)}, \ldots ,H_{i - 1}^{\left( 2 \right)},H_{i + 1}^{(2)} \ldots ,H_{M + 1}^{(2)}} \}$. We use $\left\{ . \right\}$ to denote a bag since the order in which examples appear in the sequence will not have any impact on the non-conformity score *η*_*i*_. In this work, the non-conformity measure is computed as follows:

3 $$\upeta _{\mathrm{i}} = A\left( {\left\{ {H_1^{\left( 2 \right)}, \ldots ,H_{i - 1}^{\left( 2 \right)},H_{i + 1}^{\left( 2 \right)} \ldots ,H_{M + 1}^{\left( 2 \right)}} \right\},H_i^{\left( 2 \right)}} \right) = \mathop {\sum}\nolimits_{j = 1,j \ne i}^{M + 1} { - \frac{{H_i^{\left( 2 \right)}.H_j^{\left( 2 \right)}}}{{\left\| {H_i^{\left( 2 \right)}} \right\|\left\| {H_j^{\left( 2 \right)}} \right\|}}}$$

The *p-value* of $H_{M + 1}^{(2)}$ w.r.t the two conformal sets can now be calculated as follows:

4 $$p( {H_{M + 1};\tau ^{septic}} ) = \frac{{\# \{ {i = 1,2,3,...,M:\eta _{i,septic} \ge \eta _{M + 1}} \}}}{M}$$

5 $$p( {H_{M + 1};\tau ^{non - septic}} ) = \frac{{\# \{ {i = 1,2,3,...,M:\eta _{i,non - septic} \ge \eta _{M + 1}} \}}}{M}$$

If conformity of the test example is achieved with either of the conformal sets at a given significance level *ε*, i.e., $p(H_{M + 1};\tau ^{septic}) \,>\, \varepsilon$ or $p(H_{M + 1};\tau ^{non - septic}) > \varepsilon$, the test example $H_{M + 1}^{(2)}$is passed onto the sepsis predictor to make a prediction. We choose *ε* to be 0.05 in our analysis, which translates to 95% confidence that the new test example $H_{M + 1}^{(2)}$ is not likely to be non-conformant to the conformal sets *τ*^*septic*^ or *τ*^*non-septic*^.

### Statistical methods and hypothesis testing

For all continuous variables, we have reported median ([25th–75th percentile]). For binary variables, we have reported percentages. The area under receiver operating characteristic (AUC) curves statistics, specificity (SPC), positive predictive value (PPV), negative predictive value (NPV), and diagnostic odds ratio (DOR) at a fixed threshold (corresponding to 80% sensitivity level on the development cohort) were calculated to measure the performance of the models. COMPOSER was designed as a notification-only tool to predict onset time of sepsis 4 h in advance (‘ideal prediction window’), no earlier than 48 h in advance (‘acceptable prediction window’) and under a silencing policy (aka, ‘snooze’^34^). Specifically, AUCs were reported under an end-user clinical response policy in which alarms fired up to 48 h prior to onset of sepsis were considered as true alarms, and the model was silenced for 6 h after an alarm was fired. Additionally, we have reported false alarms per patient hour (FAPH) which can be used to calculate the expected number of false alarms per unit of time in a typical care unit (e.g., a FAPH of 0.025 translates to roughly 1 alarm every 2 h in a 20-bed care unit). The FAPH was calculated by dividing the total number of false alarms by the total number of data points (sum of hourly time points across all patients) in a given cohort. Statistical comparison of all AUC curves was performed using the method of DeLong et al.^49^ Statistical comparison of DOR was performed using the paired t-test. Additionally, as a secondary end-point we considered the time from model prediction (when the risk score crosses the prediction threshold) to sepsis for the ED and ICU patient populations, since it’s possible to have a sepsis prediction model with high performance but with no lead time (for instance by incorporating treatment information as a feature in the model).

Given that the COMPOSER algorithm was designed to be generalizable, we postulated that sensitivity and specificity of the COMPOSER score would not vary significantly, assuming that the physiological characteristics of septic compared to non-septic patients would remain similar, across the various cohorts. Based on preliminary observations from our development dataset we anticipated that at 80% sensitivity COMPOSER would achieve at least 90% specificity across various cohorts. Additionally, we observed a sepsis (hourly window-wise) incidence rate of roughly 3.0–4.0% in the ICU and 2.0–5.5% in the ED. Plugging these incident rates into the standard equations for PPV (and NPV) as a function of sensitivity and specificity yields approximate values in the range of 20.0–25.0% (and NPV of 99.0–99.3%) in the ICU and 14.0–30.0% (and NPV of 98.7–99.5%) in the ED, respectively. As such, we formed two hypotheses for the ICU and ED populations as follows. We hypothesized that across the various ICU patient cohorts, with 95% power (beta = 0.05; type-II error) at an alpha of 5% (type-I error) with sufficient sample size^50^, we can show that the COMPOSER score achieves at least 20% PPV and 98% NPV. Similarly, we hypothesized that across the various ED patient cohorts, with 95% power (beta=0.05; type-II error) at an alpha of 5% (type-I error) with sufficient sample size we can show that the COMPOSER score achieves at least 10% PPV and 98% NPV. (Please see Supplementary Note 8 for sample size calculations). Additionally, we hypothesized that COMPOSER yields higher AUC, PPV, NPV, and DOR compared to competing models (GB-Vital, baseline FFNN, and ESPM models) as described next. All baseline models (including GB-Vital) were trained using the same sepsis criteria for consistency. The only exception was the ESPM risk model, since we only had access to the ESPM risk scores in our electronic health record (not the actual model).

#### GB-Vital

This is a replication of the sepsis detection model as proposed by Mao et al.^6,15^.The model corresponds to a gradient-boosted classifier of decision trees built using six vital signs measurements: systolic blood pressure, diastolic blood pressure, heart rate, respiratory rate, oxygen saturation, and temperature.

#### FFNN

This model corresponds to a 2 layer feedforward neural network (of size 40 and 25) that uses the same number of input features as that of COMPOSER.

#### ESPM

This model corresponds to the Epic’s commercially available Best Practice Advisory (BPA) alert^51^. We only had access to the risk scores produced by this system at Hospital-A during the temporal validation time-frame.

### Data processing, training, and hyperparameters

First, the combined Hospital-A ICU and ED training set was standardized by first applying normalization transformations, followed by subtracting the mean and dividing by the standard deviation. Next, all remaining datasets were normalized using exactly the same transformations utilized in the training data. For handling missing data, we used a simple sample-and-hold approach in all the datasets, with mean-imputation at the start of all time series records.

#### Weighted input layer

The scaling factors *α*_*i*_ were all initialized to 1. Model: The learning rates for encoder, sepsis predictor, and domain classifier were set to 0.01. To minimize overfitting and to improve generalizability of the model, L1–L2 regularization was used with L2 regularization parameter set to 1e-3 for encoder and sepsis predictor, 1e-4 for domain classifier and L1 regularization parameter set to 1e-3 for encoder and sepsis predictor, 1e-4 for domain classifier. Mini-batch size for the source dataset was fixed at a total of 10000 windows (50% septic windows, 50% non-septic windows). Mini-batch size for the target dataset was set at 5000 windows. The encoder, sepsis predictor, and domain classifiers were each composed of a single layer neural network of dimensions 40, 25, and 25 neurons, respectively. Both the sepsis predictor and domain classifier were further followed by a fully connected layer and a softmax layer. Conformal predictor: Threshold *ε* was set at 0.05. The conformal set consisted of an equal proportion of septic windows and non-septic windows, optimized to minimize the deleterious effects of examples with large numbers of missingness on prediction performance. COMPOSER was trained for a total of 500 epochs using Adam optimizer^52^, with early stopping. All hyper-parameters of the model (number and size of layers for encoder-sepsis predictor-domain classifier, learning rate, mini-batch size, L1 regularization parameter, and L2 regularization parameter) were optimized using Bayesian optimization on the validation set of the development site^53^. All pre-processing of data was performed using Numpy^54^ with the rest of the pipeline implemented using TensorFlow^55^.

### Reporting summary

Further information on research design is available in the Nature Research Reporting Summary linked to this article.

## Supplementary information

Supplementary Information

Reporting Summary

## Data Availability

A sample dataset containing: (1) predicted risk scores of 500 patients from COMPOSER and (2) code for computing performance metrics is available at https://github.com/NematiLab/COMPOSER. De-identified data from the Emory cohort has been made available as part of the PhysioNet Challenge 2019 (https://physionet.org/content/challenge-2019/1.0.0/). Access to de-identified UCSD cohort may be made available by contacting the corresponding author and via approval from UCSD Institutional Review Board (IRB) and Health Data Oversight Committee (HDOC).

## Code availability

Access to the computer code used in this research is available upon request to the corresponding author.
