# Supplementary material for: Artificial intelligence sepsis prediction algorithm learns to say “I don’t know”
Source: NPJ Digit Med. 2021 Sep 9;4:134. doi: 10.1038/s41746-021-00504-6 (PMC8429719; doi:10.1038/s41746-021-00504-6)
Supplement: Supplementary file 1 — Supplementary Information [file 41746_2021_504_MOESM1_ESM.pdf]

Supplementary Material for: Artificial Intelligence Sepsis Prediction Algorithm Learns to Say “I don’t know”

Supreeth P. Shashikumar, PhD[1*], Gabriel Wardi, MD, MPH[2,3], Atul Malhotra, MD[3], Shamim Nemati, PhD[1*]

[1]Division of Biomedical Informatics, University of California San Diego, San Diego, USA
[2]Department of Emergency Medicine, University of California San Diego, San Diego, USA
[3]Division of Pulmonary, Critical Care and Sleep Medicine, University of California San Diego, San Diego, USA

[*]To whom correspondence should be addressed; E-mail: spshashikumar, snemati@health.ucsd.edu

Supplementary Note 1. Clinical variables for model development

Supplementary Table 1: List of clinical variables

Variable	Measurement Unit	Variable	Measurement Unit
Vital Signs (Dynamical Features)			
Heart rate	*beats/minute*	Mean Arterial Pressure	*mmHg*
Pulse oximetry	*%*	Diastolic BP	*mmHg*
Temperature	*degC*	Respiration rate	*breaths per minute*
Systolic BP	*mmHg*	End tidal CO_2	*mmHg*
Laboratory values (Dynamical Features)			
Excess bicarbonate	*mmol/L*	Serum Glucose	*mg/dL*
Bicarbonate	*mmol/L*	Lactic acid	*md/dL*
Fraction of inspired Oxygen	*%*	Magnesium	*mmol/dL*
pH	-	Phosphate	*mg/dL*
Partial pressure of CO_2 from arterial blood	*mmHg*	Potassium	*mmol/L*
Oxygen saturation from arterial blood	*%*	Total Bilirubin	*mg/dL*
Aspartate transaminase	*IU/L*	Troponin I	*ng/mL*
Blood Urea Nitrogen	*mg/dL*	Hematocrit	*%*
Alkaline phosphate	*IU/L*	Hemoglobin	*g/dL*
Calcium	*mg/dL*	Partial Thromboplastin Time	*seconds*
Chloride	*mmol/L*	White Blood Cell count	*count*$10^3/\mu L$*
Creatinine	*mg/dL*	Fibrinogen	*mg/dL*
Bilirubin direct	*mg/dL*	Platelets	*count*$10^3/\mu L$*
Demographics			
Age	*Years*	Hours between hospital admit and care unit admit	*hours*
Gender	*Male/Female*	Duration until current time	*hours*
Care Units	*Medical/Surgical ICU unit*	--	--

Supplementary Note 2. Patient characteristics

Supplementary Tables 2 and 3 provides a summary of patient characteristics of the ICU and ED cohorts University of California San Diego Health (including two hospitals collectively termed as Hospital-A) and Emory University Hospital (including four hospitals collectively termed as Hospital-B) considered in this study.

Supplementary Table 2: Characteristics of septic and non-septic population in the three ED cohorts.

	**Hospital-A ED**		**Hospital-A Temporal ED**		**Hospital-B ED**	
	Non-septic	Septic	Non-septic	Septic	Non-septic	Septic
No. Patients, *n (%)*	90,905	8,130 (8.2%)	18,148	1,797 (9.0%)	315,845	14,454 (4.4%)
Age (yrs), *median [IQR]*	55.1 [39.6 66.6]	61.2 [48.9 72.4]	56.7 [40.3 68.4]	62.1 [48.6 71.7]	51 [34 67]	64 [50 76]
Male, *n (%)*	48,816 (53.7%)	4,602 (56.6%)	9,782 (53.9%)	1,019 (56.7%)	125,390 (39.7%)	7,169 (49.6%)
Race, *n (%)*						
Caucasian	49,271 (54.2%)	4,325 (53.2%)	9,473 (52.2%)	888 (49.4%)	148,763 (47.1%)	7,025 (48.6%)
African American	12,272 (13.5%)	813 (10.0%)	2,305 (12.7%)	165 (9.2%)	149,710 (47.4%)	6,591 (45.6%)
Asian	5,091 (5.6%)	626 (7.7%)	1,016 (5.6%)	147 (8.2%)	7,580 (2.4%)	390 (2.7%)
ED LOS (hrs), *median [IQR]*	8.3 [6.0 13.4]	9.2 [6.6 15.1]	8.1 [5.7 12.8]	9.3 [6.7 16.3]	6.7 [5.1 9.5]	6.9 [5.4 9.3]
CCI, *median [IQR]*	3 [2 7]	5 [3 9]	3 [1 6]	5 [3 8]	0 [0 1]	3 [2 5]
SOFA (yrs), *median [IQR]*	0 [0 1]	2 [1 3]	0 [0 1]	2 [1 4]	0 [0 1]	3 [2 4]
ED Admission to t_{sepsis} (hrs),	-	3.8 [2 6.1]	-	3.4 [2 5.6]	-	3.5 [2.3 5.6]
Inpatient mortality, *n (%)*	454 (0.5%)	545 (6.7%)	91 (0.5%)	113 (6.3%)	631 (0.2%)	622 (4.3%)
Inpatient hospice, *n (%)*	181 (0.2%)	65 (0.8%)	18 (0.1%)	15 (0.8%)	947 (0.3%)	694 (4.8%)

Supplementary Table 3: Characteristics of septic and non-septic population in the three ICU cohorts.

	**Hospital-A ICU**		**Hospital-A Temporal ICU**		**Hospital-B ICU**	
	Non-septic	Septic	Non-septic	Septic	Non-septic	Septic
No. Patients, *n (%)*	13,208	3,825 (22.5%)	2,863	733 (20.4%)	37,899	7,913 (17.3%)
Age (yrs), *median [IQR]*	60.2 [46.8 71.3]	60.7 [48.1 72.2]	61.5 [46.7 70.6]	61.0 [47.7 69.9]	62 [50 72]	62 [50 72]
Male, *n (%)*	7,978 (60.4%)	2,463 (64.4%)	1,732 (60.5%)	466 (63.6%)	20,313 (53.6%)	4,305 (54.4%)
Race, *n (%)*						
Caucasian	7,013 (53.1%)	1,897 (49.6%)	1,466 (51.2%)	319 (43.5%)	21,084 (55.1%)	1,153 (49.1%)
African American	977 (7.4%)	295 (7.7%)	212 (7.4%)	64 (8.7%)	18,583 (36.6%)	1,164 (42.1%)
Asian	713 (5.4%)	241 (6.3%)	169 (5.9%)	56 (7.7%)	561 (2.5 %)	33 (2.1%)
ICU LOS (hrs), *median [IQR]*	45.6 [25.8 80.9]	188.7 [93.4 381.3]	48.9 [27.9 90.4]	206.9 [103.7 383.5]	45.2 [25.5 75.6]	176.3 [87.2 353.1]
CCI, *median [IQR]*	3 [1 6]	4 [2 7]	3 [1 6]	4 [2 7]	3 [1 5]	4 [2 6]
SOFA (yrs), *median [IQR]*	3 [1 5]	7 [4 10]	3 [1 5]	7 [4 9]	2 [1 5]	7 [4 10]
ICU Admission to t_{sepsis} (hrs),	-	22.6 [11.2 52.6]	-	23.2 [10.6 59.2]	-	26.3 [10.3 67.3]
Inpatient mortality, *n (%)*	357 (2.7%)	845 (22.1%)	83 (2.9%)	136 (18.6%)	872 (2.3%)	1,282 (16.2%)
Inpatient hospice, *n (%)*	53 (0.4%)	31 (0.8%)	3 (0.1%)	5 (0.7%)	682 (1.8%)	847 (10.7%)

Supplementary Note 3. Summary of COMPOSER performance on the development cohort training set

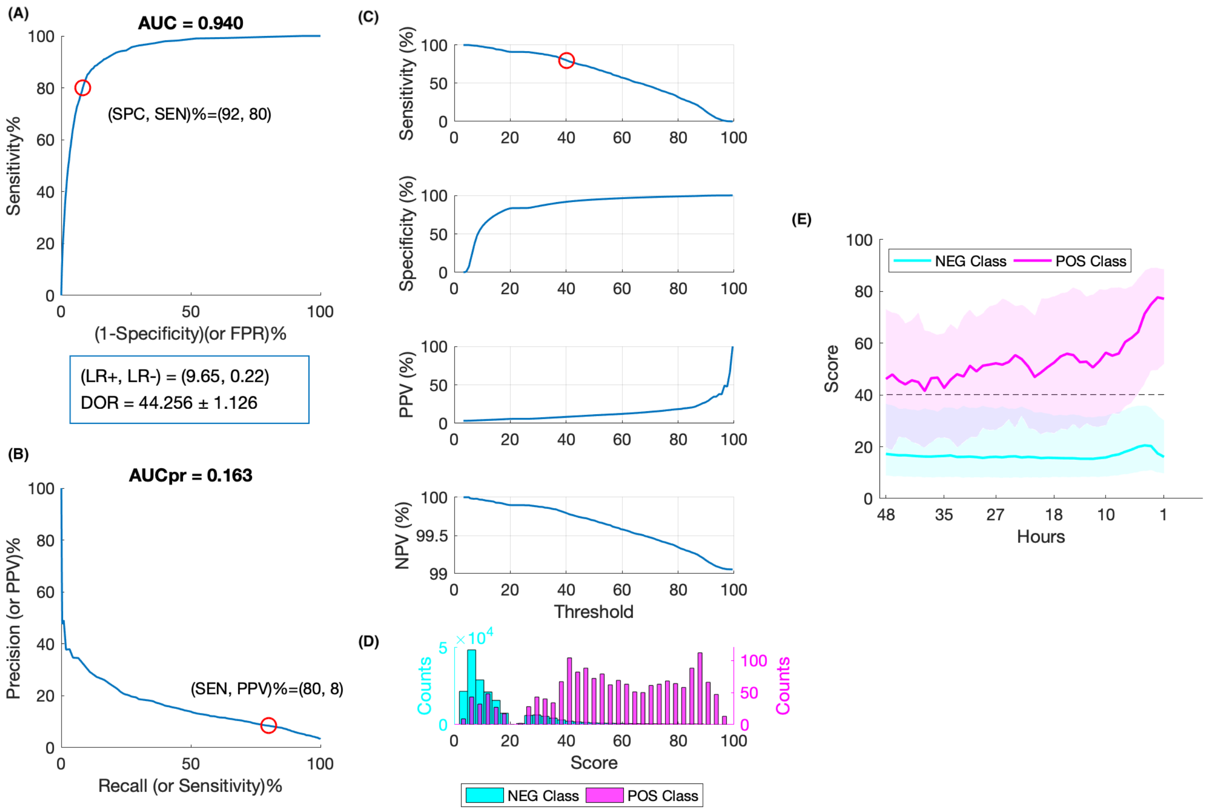

Supplementary Figure 1: The area under the receiver operating characteristic curve (AUC) and Area under the precision recall curve (AUCpr) plots of COMPOSER on the combined Hospital-A ICU and cohort are shown in Panels (A) and (B) respectively. The Sensitivity, Specificity, Positive predictive value and Negative predictive value plots as a function of risk score threshold is shown in Panel (C). The histogram plots of all non-septic samples (NEG class) and septic samples (POS class) are shown in Panel (D). The median [IQR] of predicted risk scores starting from 48 hours prior and until onset of sepsis (for septic patients) or until end of patient record (for non-septic patients) are shown in Panel (E).

Supplementary Note 4. Performance of COMPOSER at patient-level

Supplementary Table 4: Summary of performance of COMPOSER when evaluated at a patient-level on the ICU cohorts. Decision threshold based on training data corresponding to 80% sensitivity level on **hourly window-wise** data.

Dataset	SEN	SPC	PPV	NPV
**Hospital-A ICU test**	91.6%	65.4%	44.0%	96.3%
**Hospital-A Temporal ICU**	92.3%	64.6%	40.2%	97.0%
**Hospital-B ICU**	91.3%	49.9%	25.9%	96.4%

SEN: Sensitivity, **SPC**: Specificity, **PPV**: Positive Predictive Value, **NPV**: Negative Predictive Value

Supplementary Table 5: Summary of performance of COMPOSER when evaluated at a patient-level on the ED cohorts. Decision threshold based on training data corresponding to 80% sensitivity level on **hourly window-wise** data.

Dataset	SEN	SPC	PPV	NPV
**Hospital-A ED test**	95.6%	69.8%	21.7%	99.5%
**Hospital-A Temporal ED**	96.0%	67.7%	22.4%	99.4%
**Hospital-B ED**	90.5%	73.9%	13.4%	99.4%

SEN: Sensitivity, **SPC**: Specificity, **PPV**: Positive Predictive Value, **NPV**: Negative Predictive Value

Supplementary Table 6: Summary of performance of COMPOSER when evaluated at a patient-level on the ICU cohorts. Decision threshold based on training data corresponding to 80% sensitivity level on **patient-wise** data.

Dataset	SEN	SPC	PPV	NPV
**Hospital-A ICU test**	81.2%	80.1%	52.9%	93.4%
**Hospital-A Temporal ICU**	82.1%	78.2%	49.1%	94.4%
**Hospital-B ICU**	82.6%	63.5%	31.4%	94.7%

SEN: Sensitivity, **SPC**: Specificity, **PPV**: Positive Predictive Value, **NPV**: Negative Predictive Value

Supplementary Table 7: Summary of performance of COMPOSER when evaluated at a patient-level on the ED cohorts. Decision threshold based on training data corresponding to 80% sensitivity level on **patient-wise** data.

Dataset	SEN	SPC	PPV	NPV
**Hospital-A ED test**	83.6%	90.0%	42.3%	98.4%
**Hospital-A Temporal ED**	84.1%	89.9%	43.7%	98.3%
**Hospital-B ED**	70.8%	92.4%	28.6%	98.4%

SEN: Sensitivity, **SPC**: Specificity, **PPV**: Positive Predictive Value, **NPV**: Negative Predictive Value

Supplementary Note 5. Analysis of false alarms

Results presented in Supplementary Table 8 show that 57.0%–66.8% and 49.5%–19 59.6% of false alarms in the ICU and ED were triggered on patients who satisfied at least one of 20 the following six conditions: 1) presence of clinical suspicion of infection without evidence of 21 acute organ dysfunction, 2) need for vasopressors, 3) requiring mechanical ventilation, 4) at risk 22 for acute kidney injury (AKI), 5) eventual transition to sepsis, but not within 48 hours of the alarms 23 and 6) at risk for mortality and/or hospice care.

Supplementary Table 8: Characteristics of the false alarm group across the three ICU and ED cohorts. Percentages (%) and cumulative percentages (Cum.) of events in the 72 hours interval following an alarm ([t_{alarm}, t_{alarm}+72] hours) by COMPOSER.

	Hospital-A ICU		Hospital-A Temporal ICU		Hospital-B ICU		Hospital-A ED		Hospital-A Temporal ED		Hospital-B ED	
	%	Cum.	%	Cum.	%	Cum.	%	Cum.	%	Cum.	%	Cum.
ABX/Cult	46.0	46.0	45.9	45.9	42.3	42.3	31.3	31.3	31.4	31.4	39.3	39.3
Pressor	23.9	53.6	11.5	48.8	12.0	44.8	1.3	31.6	0.7	31.6	0.5	39.4
Mech Vent	23.1	57.4	24.2	54.4	17.3	47.8	1.5	31.9	1.4	31.9	1.8	39.9
AKI	9.6	59.7	6.1	56.1	10.8	50.8	22.9	48.2	24.5	49.1	29.9	59.1
T0 event	44.4	64.7	40.9	59.9	26.6	53.7	4.4	48.9	4.3	49.8	4.4	59.3
Expired	20.3	66.8	15.0	62.9	10.1	55.4	1.6	49.3	1.5	50.0	0.8	59.4
Hospice	1.4	**66.8**	0.1	**63.1**	5.6	**57.0**	0.3	**49.5**	0.2	**50.1**	1.2	**59.6**

Tsusp: suspicion of infection (t_{alarm}+72 hours); **Pressor**: need for pressors (t_{alarm}+72 hours);
Mech Vent: need for 16 mechanical ventilation (t_{alarm}+72 hours); **AKI**: acute kidney injury (t_{alarm}+72 hours);
T0 event: eventual development of sepsis; **Expired**: eventual hospital death;
Hospice: eventual transition to hospice; **Cum**: cumulative sum of conditions, indicating one or more of the conditions

Supplementary Note 6. Sepsis prediction performance improves with weighted input layer

Supplementary Table 9: Comparison of performance of baseline models (AUC) trained on Hospital-A ICU and ED cohorts.

**Dataset**	**FFNN**[1] (AUC/PPV/SPC)	**FFNN**[2] **with weighted input layer** (AUC/PPV/SPC)
**Hospital-A ICU**	0.932/36.4%/74.2%	0.952/34.0%/92.9%
**Hospital-A Temporal ICU**	0.931/34.3%/76.4%	0.953/32.0%/93.6%
**Hospital-B ICU**	0.910/23.0%/67.0%	0.925/21.7%/89.9%
**Hospital-A ED**	0.930/18.0%/76.8%	0.937/15.5%/91.5%
**Hospital-A Temporal ED**	0.926/19.1%/73.0%	0.931/16.0%/90.0%
**Hospital-B ED**	0.910/13.0%/82.1%	0.934/10.0%/93.1%

[1] *Input =* $[X_{dyamical}; X_{covar}; X_{TSLM}]$.
[2] *FFNN with weighted input layer.* X_{TSLM} *used only to scale* $X_{dyamical}$*, and not used as input feature to FFNN.*
[3] *Input =* $[X_{dyamical}; X_{covar}]$.

Due to workflow-related variations in frequency of laboratory measurements in ICUs and EDs, we hypothesized that when trained on ICU and ED data, a FFNN with weighted input layer (in this model X_{TSLM} is used to only scale $X_{dynamical}$, and not used as input feature to FFNN) would outperform a FFNN (input = $[X_{dyamical}; X_{covar}; X_{TSLM}]$) which has access to the missingness data directly as an input feature (and thus likely to overfit to the ICU workflow processes and patterns of data missingness).

The data in Supplementary Table 9 show that the FFNN with weighted input layer performed significantly better when compared to a baseline FFNN.

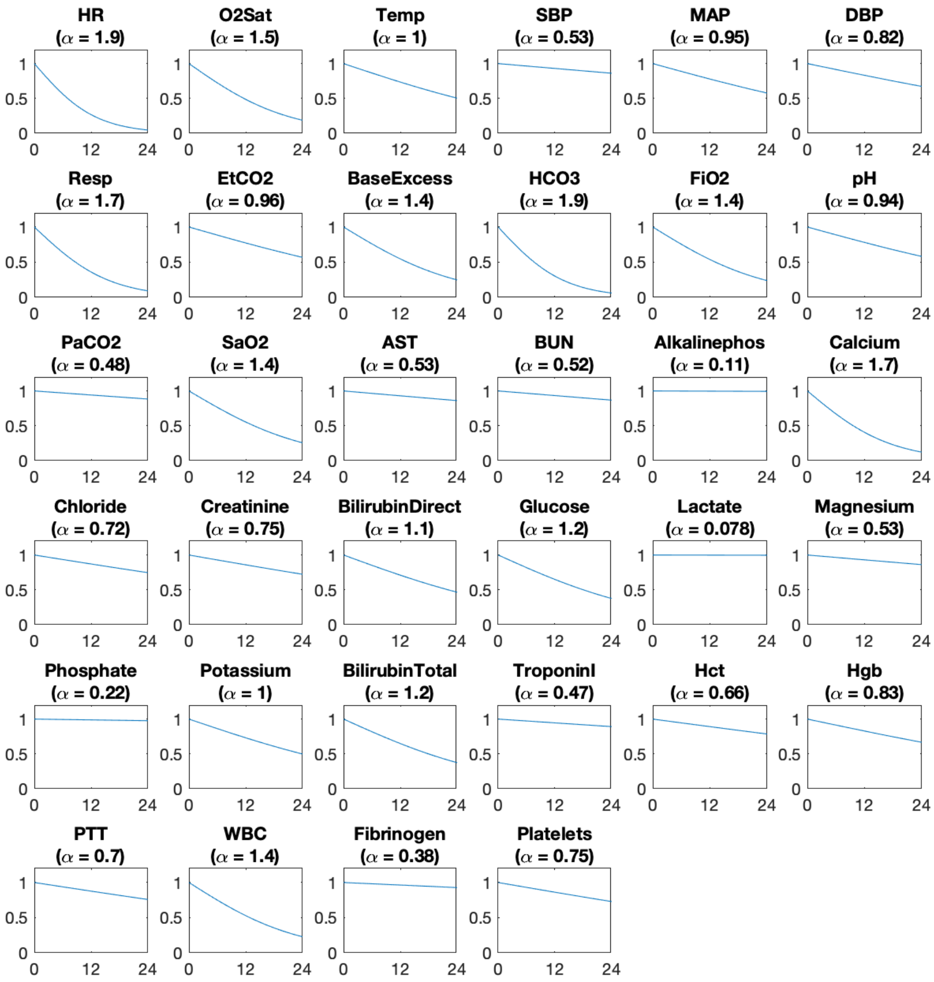

Supplementary Figure 2: Illustration of the weighting scheme learnt by COMPOSER trained on Hospital-A ICU and ED cohorts. The plots shown depict the scaling function (varies from 0 to 1) imposed by the model for various values of Time Since Last Measurement (varies from 0 to 24 hours) of each of the 34 dynamical variables considered in our study.

Supplementary Note 7. Detecting distribution shift using conformal prediction

Supplementary Note 7(A): When does conformal prediction accept or reject samples?

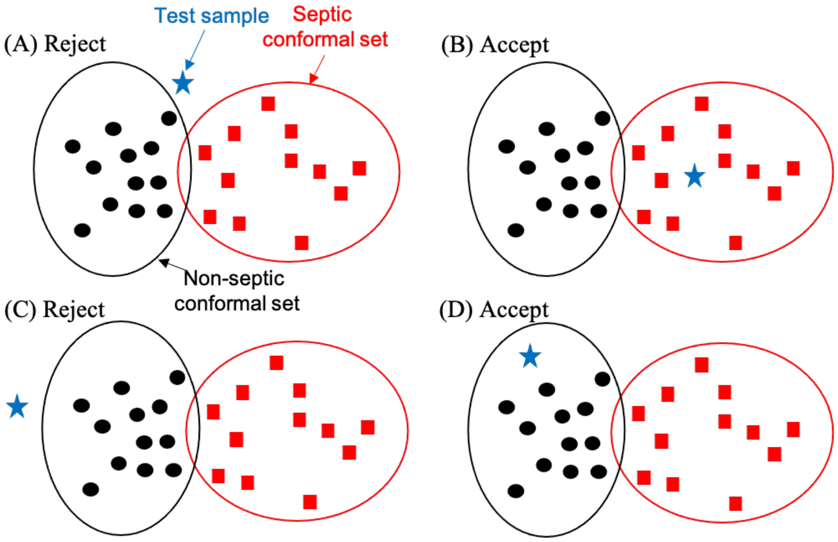

Supplementary Figure 3: Panels (A) and (C) show scenarios under which a test sample is situated outside the boundaries of the septic and non-septic conformal sets as a result of which are rejected by conformal prediction. Panels (B) and (D) show scenarios under which a test sample is situated within the septic or non-septic conformal set as a result of which are accepted by conformal prediction.

Supplementary Note 7(B): What are the rejection rates within each of the cohorts?

Supplementary Table 10 provides percentages of rejected windows. Since we make predictions in a sequential setting (from time of admission to time T0 of sepsis, divided into hourly time bins) with a fixed prediction horizon (e.g., 4-6 hours), a given septic patient record may include several non-septic labels prior to onset time of sepsis. As such, one may only focus on the septic labels and analyze the rejection rate of the conformal prediction on those time bins with positive labels. Supplementary Table 11 provides rejection statistics for the entire record as well as ‘only septic windows’, indicating that the overall rejection of septic cases is very low.

Supplementary Table 10: % of datapoints rejected across the entire cohort by the conformal prediction module when COMPOSER was used for prediction.

**Dataset**	**Overall** (Non-septic/Septic)
Hospital-A ICU	27.8% (27.9%/13.2%)
Hospital-A ED	14.3% (14.7%/6.5%)
Hospital-A Temporal ICU	27.6% (28.1%/12.4%)
Hospital-A Temporal ED	16.1% (16.6%/6.8%)
Hospital-B ICU	24.6% (24.9%/13.9%)
Hospital-B ED	13.6% (13.7%/9.9%)

Supplementary Table 11: % of datapoints (or windows) rejected per-patient within each of the six cohorts when COMPOSER was used for prediction.

**Dataset**	**Non-septic patients** median [IQR]	**Septic patients** median [IQR]	
	Entire record considered	Only Septic windows considered	Entire record considered
Hospital-A ICU	29.7% [11.6%, 40.6%]	0% [0%, 14.2%]	13.3% [2.6%, 36.4%]
Hospital-A ED	4.5% [0%, 25%]	0% [0%, 0%]	0% [0%, 0%]
Hospital-A Temporal ICU	27.3% [13.8%, 47.5%]	0% [0%, 14.3%]	11.9% [2.1%, 20.7%]
Hospital-A Temporal ED	11.1% [0%, 33.3%]	0% [0%, 0%]	0% [0%, 0%]
Hospital-B ICU	24.4% [10.5%, 40.6%]	0% [0%, 14.2%]	15.1% [4.1%, 32.8%]
Hospital-B ED	5.3% [0%, 28.6%]	0% [0%, 0%]	0% [0%, 12.5%]

Supplementary Note 7(C): What proportion of examples within the accepted and rejected group are false positives?

Results presented in Supplementary Table 12 show that the false positive rate within the rejected group is about 5 times higher compared to the accepted group in the Hospital-B ICU and ED cohorts. It can be inferred that conformal prediction was able to reject a higher proportion of examples that would have potentially been false alarms.

Supplementary Table 12: Summary of false positive rates within the accepted and rejected groups.

**Dataset**	**False positive rate within accepted group**	**False positive rate within rejected group**
Hospital-B ICU test	18.9%	90.4%
Hospital-B ED test	17.8%	91.1%

Supplementary Note 8. Optimal sample size for computing PPV and NPV

The optimal sample sizes were derived for 95% power (beta=0.05; type-II error) at an alpha of 5% (type-I error), sensitivity of 80% and specificity of 90% based on formulae derived in Steinberg et al. [1]. To power both PPV and NPV calculations we use the maximum sample size required to test the hypothesis for PPV and NPV calculations, as described in Eqns. (3.6) and (3.8) of Steinberg et al. [1], respectively.

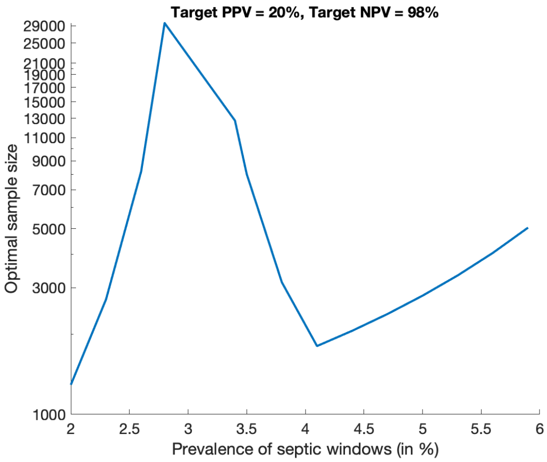

Supplementary Figure 4: The optimal sample size required for a target PPV of 20% and NPV of 98%

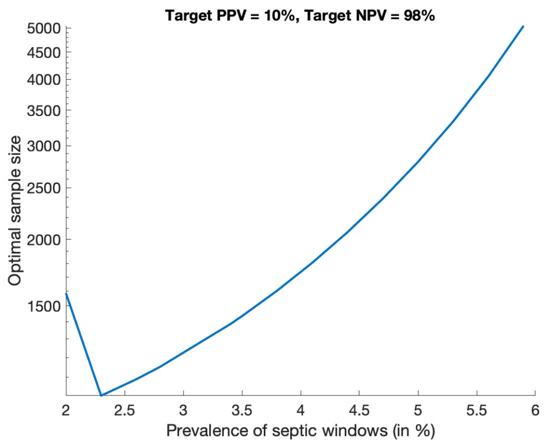

Supplementary Figure 5: The optimal sample size required for a target PPV of 10% and NPV of 98%

Supplementary Note 9. Is COMPOSER interpretable?

COMPOSER is uniquely interpretable wherein apart from computing the risk score, the model identifies the most relevant features contributing to the risk score as well. The importance of each feature's contribution to the risk score is measured through a metric called *relevance score*. To compute the relevance score, we simply take the derivative (or gradient) of the risk score with respect to all input features and multiply it by the input features. The relevance score simply says that an input feature is relevant if it is both present in the data and if the model reacts to it (the derivative term). Additionally, the direction of influence of a variable on the increase in risk score can be deduced from the sign of the input gradients. In our analysis, we only extract the top contributing features with a positive relevance score.

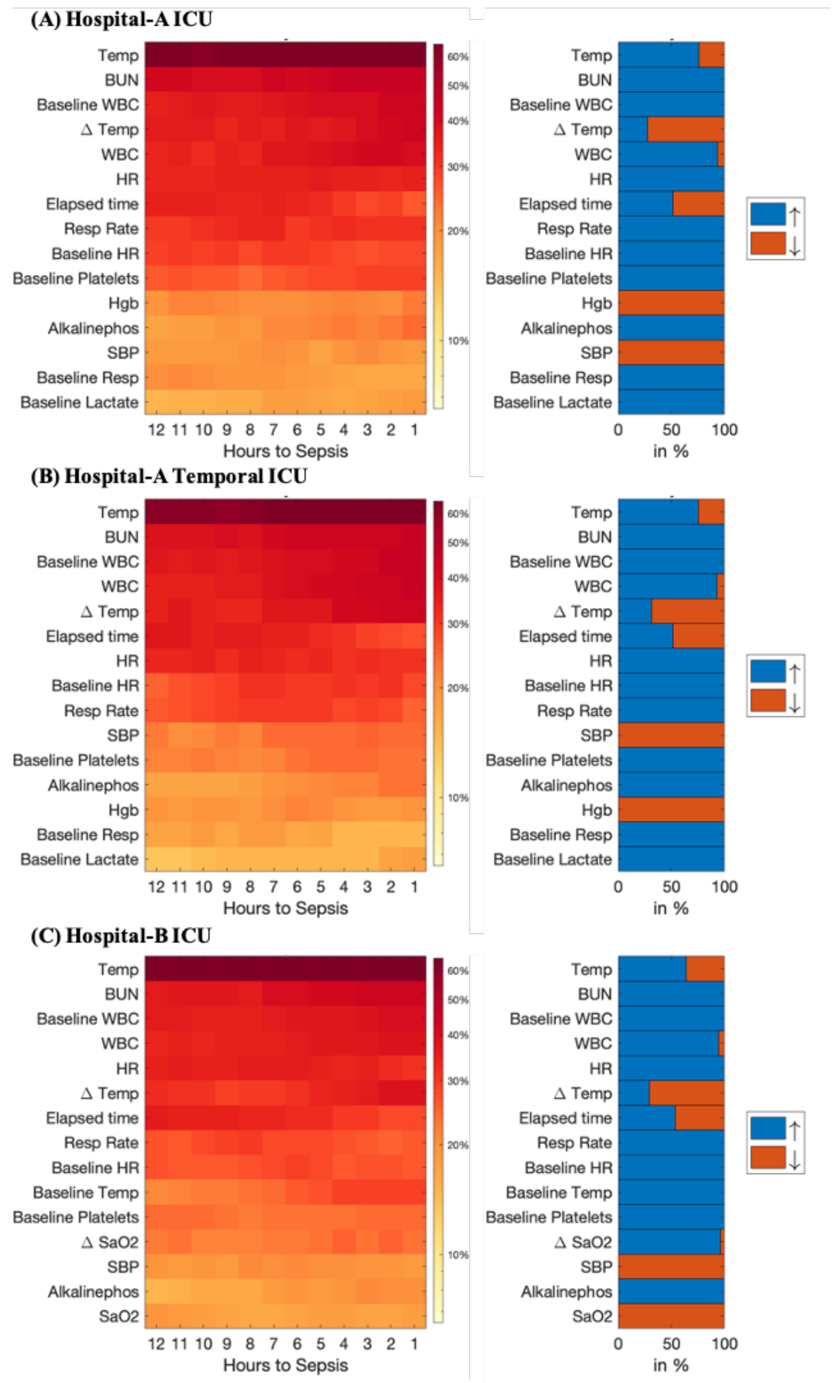

Supplementary Figure 6: Population-level plot of top contributing factors to the increase in model risk score for (A) Hospital-A ICU, (B) Hospital-A Temporal ICU, and (C) Hospital-B ICU cohorts. For each cohort, we show a heatmap (left panel) and a directionality plot (right panel). Details of heatmap: The x-axis represents hours prior to onset time of sepsis. The y-axis represents the top factors sorted by the magnitude of relevance score. The heat-map shows the percentage of septic patients for whom a given variable was an important contributor to their risk score, up to 12 hours prior to onset of sepsis. Details of directionality plot: Directionality with respect to influence of top factors contributing to an increase in the risk score. The proportion of instances in which a delta increase in values of a feature contributed to an increase in risk score is represented in blue, and the proportion of instances in which a delta decrease in values of a feature contributed to an increase in risk score is represented in red.

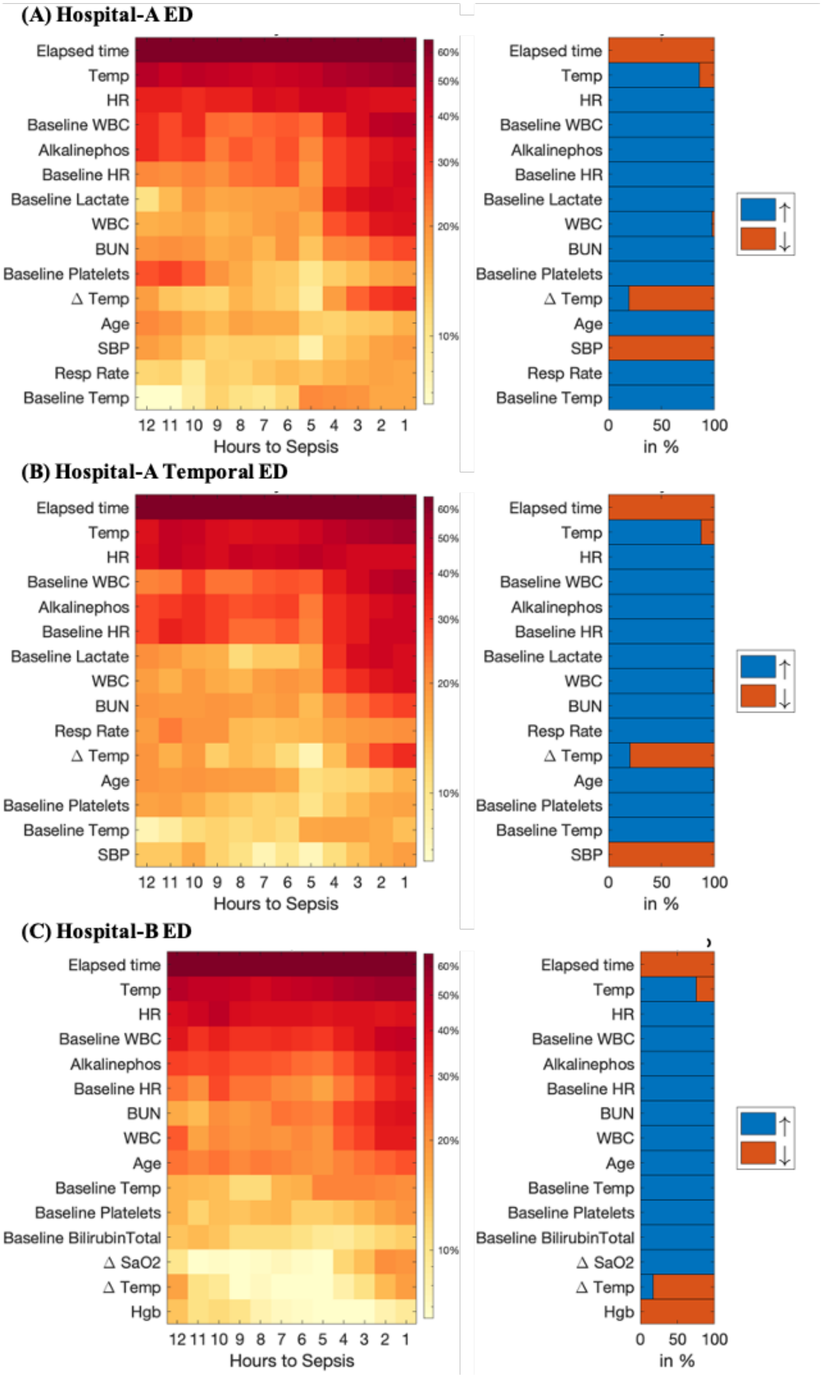

Supplementary Figure 7: Population-level plot of top contributing factors to the increase in model risk score for (A) Hospital-A ED, (B) Hospital-A Temporal ED, and (C) Hospital-B ED cohorts. For each cohort, we show a heatmap (left panel) and a directionality plot (right panel).

Supplementary Note 10. COMPOSER Software Pipeline

COMPOSER software was specifically designed to ensure interoperability to enable EHR-agnostic real-time deployment. The COMPOSER software includes two main components: 1) a containerized deep learning-based sepsis prediction algorithm ("COMPOSER microservice"), which takes the form of a TensorFlow-based executable and handles data transformation and conformal prediction; and 2) API wrappers which facilitate pulling of algorithm inputs (via FHIR calls), data cleaning, feature calculations, calling of the COMPOSER microservice, and parsing the resulting risk scores, confidence intervals and the top contributing factors. COMPOSER specifically supports two forms of interfacing with external software:

- **Periodic Interaction:** Clinical variables required for prediction ("input data") can be obtained via periodic API calls to a FHIR server (as a JSON object), followed by sepsis risk calculations and transmission of the corresponding meta-data via an HL7 message. This mode is optimized for sending information back to electronic health records or any other approved system on the network that "listens" for incoming HL7 messages (via asynchronous HL7-V2 protocols).

- **On-Demand Interaction:** Through use of a representational state transfer ("REST") API, COMPOSER accepts a request for risk scores for one or more patients (as a JSON object) and returns the risk scores and the related meta-data as another JSON object. This mode is appropriate for interacting with external software that may require receiving the risk scores and related meta-data on a on-demand basis.

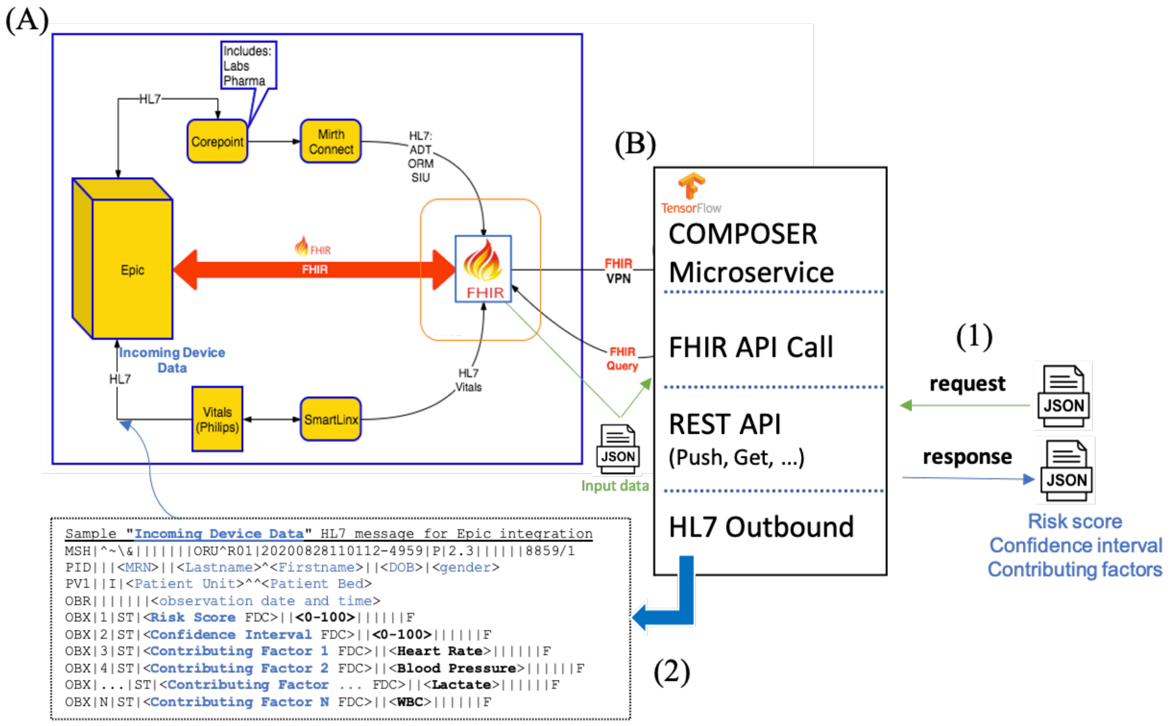

Supplementary Figure 8: Schematic diagram of the COMPOSER Software Pipeline (panel B), pictured interacting with streaming data from an electronic health record (e.g., laboratory data from Epic) and bedside monitors (e.g., vital signs from Philips monitors) harmonized in a FHIR server for interoperability (panel A). Both the “periodic” (1) and “on demand” (2) interaction modes are pictured.

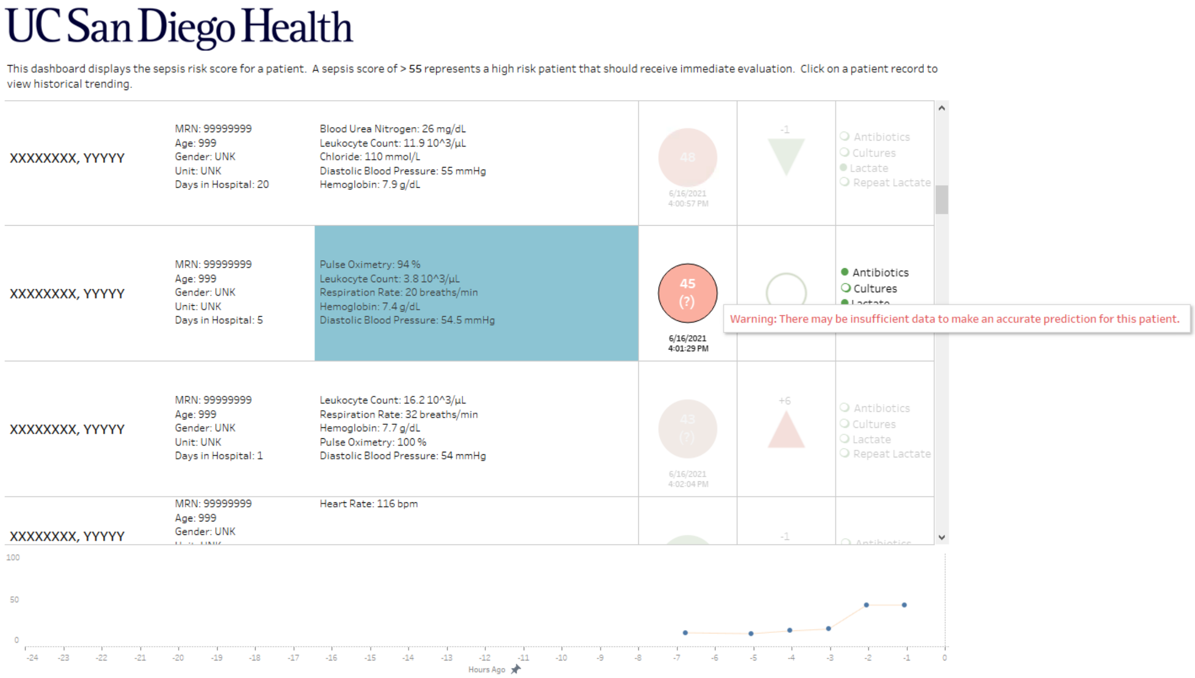

Supplementary Figure 9: The clinician facing User Interface (UI) with an indeterminate data point highlighted. An outlier focused warning message is shown alongside the risk score of a data point tagged as indeterminate.

Supplementary References

[1] Steinberg, D. M., Fine, J. & Chappell, R. Sample size for positive and negative predictive value in diagnostic research using case–control designs. *Biostatistics* **10**, 94–105 (2009).
