# Supplementary material for: Artificial intelligence sepsis prediction algorithm learns to say “I don’t know”
Source: NPJ Digit Med. 2021 Sep 9;4:134. doi: 10.1038/s41746-021-00504-6 (PMC8429719; doi:10.1038/s41746-021-00504-6)
Supplement: Supplementary file 2 — Reporting Summary [file 41746_2021_504_MOESM2_ESM.pdf]

nature research

Corresponding author(s): Supreeth P. Shashikumar, Shamim Nemati

Last updated by author(s): Jul 31, 2021

Reporting Summary

Nature Research wishes to improve the reproducibility of the work that we publish. This form provides structure for consistency and transparency in reporting. For further information on Nature Research policies, see our Editorial Policies and the Editorial Policy Checklist.

Statistics

For all statistical analyses, confirm that the following items are present in the figure legend, table legend, main text, or Methods section.

n/a	Confirmed	
☐	☒	The exact sample size (*n*) for each experimental group/condition, given as a discrete number and unit of measurement
☐	☒	A statement on whether measurements were taken from distinct samples or whether the same sample was measured repeatedly
☐	☒	The statistical test(s) used AND whether they are one- or two-sided<br>*Only common tests should be described solely by name; describe more complex techniques in the Methods section.*
☐	☒	A description of all covariates tested
☒	☐	A description of any assumptions or corrections, such as tests of normality and adjustment for multiple comparisons
☐	☒	A full description of the statistical parameters including central tendency (e.g. means) or other basic estimates (e.g. regression coefficient) AND variation (e.g. standard deviation) or associated estimates of uncertainty (e.g. confidence intervals)
☐	☒	For null hypothesis testing, the test statistic (e.g. *F*, *t*, *r*) with confidence intervals, effect sizes, degrees of freedom and *P* value noted<br>*Give P values as exact values whenever suitable.*
☒	☐	For Bayesian analysis, information on the choice of priors and Markov chain Monte Carlo settings
☒	☐	For hierarchical and complex designs, identification of the appropriate level for tests and full reporting of outcomes
☒	☐	Estimates of effect sizes (e.g. Cohen's *d*, Pearson's *r*), indicating how they were calculated

Our web collection on statistics for biologists contains articles on many of the points above.

Software and code

Policy information about availability of computer code

Data collection	The databases were queried in SQL server 2017.
Data analysis	Data pre-processing was performed using Numpy v1.15.1. All deep learning models were implemented using Tensorflow v1.12. Please see manuscript methods section for more detail

For manuscripts utilizing custom algorithms or software that are central to the research but not yet described in published literature, software must be made available to editors and reviewers. We strongly encourage code deposition in a community repository (e.g. GitHub). See the Nature Research guidelines for submitting code & software for further information.

Data

Policy information about availability of data

All manuscripts must include a data availability statement. This statement should provide the following information, where applicable:

- Accession codes, unique identifiers, or web links for publicly available datasets
- A list of figures that have associated raw data
- A description of any restrictions on data availability

De-identified data from the Emory cohort has been made available as part of the PhysioNet Challenge 2019 (https://physionet.org/content/challenge-2019/1.0.0/). Access to de-identified UCSD cohort may be made available by the corresponding authors and via approval from UCSD Institutional Review Board (IRB) and Health Data Oversight Committee (HDOC). Access to the computer code used in this research is available upon request to the corresponding author.

Field-specific reporting

Please select the one below that is the best fit for your research. If you are not sure, read the appropriate sections before making your selection.

☒ Life sciences ☐ Behavioural & social sciences ☐ Ecological, evolutionary & environmental sciences

For a reference copy of the document with all sections, see nature.com/documents/nr-reporting-summary-flat.pdf

Life sciences study design

All studies must disclose on these points even when the disclosure is negative.

Sample size	We included all adult patients from two healthcare systems in the United States across intensive care units (ICU) and emergency departments (ED)
Data exclusions	Patients 18 years or older were followed throughout their stay until development of sepsis or discharge. To allow for initial examination and stabilization of patients and adequate data collection for prediction purposes, we focused on sequential hourly prediction of sepsis starting at hours two and four within our ED and ICU cohorts, respectively. Patients who developed sepsis prior to prediction start time or those with no measurement of heart rate or blood pressure prior to the prediction start time or those whose length of stay (ICU or ED depending on the cohort) was more than 21 days were excluded.
Replication	The model testing was replicated on the external validation and temporal validation cohorts, as explained in the results and methods section.
Randomization	For Hospital-A ICU and ED cohorts, data were randomly split into training (70% of patients), hyper-parameter optimization (10% of patients) and testing (20% of patients). All data for a single patient was assigned to exactly one of these splits. (See paper methods for more detail.)
Blinding	When assigning patients randomly to test, hyper-parameter optimization and training groups investigators were blinded to patient covariates.

Reporting for specific materials, systems and methods

We require information from authors about some types of materials, experimental systems and methods used in many studies. Here, indicate whether each material, system or method listed is relevant to your study. If you are not sure if a list item applies to your research, read the appropriate section before selecting a response.

Materials & experimental systems

n/a	Involved in the study	
☒	☐	Antibodies
☒	☐	Eukaryotic cell lines
☒	☐	Palaeontology and archaeology
☒	☐	Animals and other organisms
☐	☒	Human research participants
☒	☐	Clinical data
☒	☐	Dual use research of concern

Methods

n/a	Involved in the study	
☒	☐	ChIP-seq
☒	☐	Flow cytometry
☒	☐	MRI-based neuroimaging

Human research participants

Policy information about studies involving human research participants

Population characteristics	The Hospital-A ICU and ED cohorts were drawn from the EHR of all patients admitted to two hospitals within UC San Diego Health between January 2016 and August 2019. A separate ICU and ED temporal validation cohorts were extracted at Hospital-A, spanning August 2019 through February 2020. The Hospital-B ICU and ED cohorts were drawn from the EHR data of all patients admitted to four hospitals within the Emory Healthcare System from January 2014 to December 2018. Patients were tagged for sepsis if they fulfilled the Third International Consensus Definitions for Sepsis (Sepsis-3), or any of the criteria for severe sepsis defined by the Surviving Sepsis Campaign Guidelines for Management of Severe Sepsis and Center for Medicare and Medicate SEP-1 (CMS) and the Center for Disease Control (CDC). The Hospital-A retrospective and temporal ED cohorts and the Hospital-B external ED validation cohort included a total of 99,035 (8.2% septic), 19,945 (9.0% septic), 330,299 (4.4% septic) patients, respectively. The Hospital-A retrospective and temporal ICU cohorts and Hospital-B external ICU validation cohort included a total of 17,033 (22.5% septic), 3,596 (20.4% septic), 45,812 (17.3% septic) patients, respectively. For more information please refer to Supplementary Tables S3 and S4 in the submitted supplementary material.
Recruitment	All adult patients admitted to ICU or ED at the Hospital-A and Hospital-B health systems were included for analysis.
Ethics oversight	This investigation was conducted according to UC San Diego IRB approved protocol #191098 and Emory IRB Protocol #00110675.

Note that full information on the approval of the study protocol must also be provided in the manuscript.
